# Changes in Waist Circumference and Mortality in Middle-Aged Men and Women

**DOI:** 10.1371/journal.pone.0013097

**Published:** 2010-09-30

**Authors:** Tina Landsvig Berentzen, Marianne Uhre Jakobsen, Jytte Halkjaer, Anne Tjønneland, Kim Overvad, Thorkild I. A. Sørensen

**Affiliations:** 1 Institute for Preventive Medicine, Copenhagen University Hospital, Centre for Health and Society, Copenhagen, Denmark; 2 Department of Cardiology, Center for Cardiovascular Research, Aalborg Hospital, Aarhus University Hospital, Aalborg, Denmark; 3 Department of Epidemiology, School of Public Health, Aarhus University Aarhus, Denmark; 4 The Danish Cancer Society, Institute of Cancer Epidemiology, Copenhagen, Denmark; Pennington Biomedical Research Center, United States of America

## Abstract

**Background:**

Waist circumference (WC) adjusted for body mass index (BMI) is positively associated with mortality, but the association with changes in WC is less clear. We investigated the association between changes in WC and mortality in middle-aged men and women, and evaluated the influence from concurrent changes in BMI.

**Methodology/Principal Findings:**

Data on 26,625 healthy men and women from the Danish Diet, Cancer and Health study was analyzed. WC and BMI were assessed in 1993–97 and in 1999–02. Information on mortality was obtained by linkage to the Danish central Person Register. Hazard ratios (HR) were estimated with Cox regression models. During 6.7 years of follow-up, 568 and 361 deaths occurred among men and women, respectively. Changes in WC were positively associated with mortality (HR per 5 cm for the sexes combined  = 1.09 (1.02∶1.16) with adjustment for covariates, baseline WC, BMI and changes in BMI), whereas changes in BMI were inversely associated with mortality (HR per kg/m2 for the sexes combined  = 0.91 (0.86, 0.97) with adjustment for covariates, baseline WC, BMI and changes in WC). Associations between changes in WC and mortality were not notably different in sub-groups stratified according to changes in BMI, baseline WC or when smokers or deaths occurring within the first years of follow-up were excluded.

**Conclusions/Significance:**

Changes in WC were positively associated with mortality in healthy middle-aged men and women throughout the range of concurrent changes in BMI. These findings suggest a need for development of prevention and treatment strategies targeted against redistribution of fat mass towards the abdominal region.

## Introduction

Obesity and weight gain increases the risk of premature morbidity and mortality [1]. Long-term population-based studies have, however, shown that weight loss is associated with increased mortality [2]–[5]. Occurrences of pre-existing or sub-clinical diseases and high-risk behaviors (as smoking) have been suggested to explain the increased risk associated with weight loss, but the increased risk of mortality persist after careful adjustment for confounders and exclusion of individuals with pre-existing diseases [2]–[5]. Alternatively, a recent study showed that a decline in skin-fold thickness (for a given weight loss) was associated with reduced mortality, whereas weight loss (for a given change in skin-fold thickness) was associated with increased mortality indicating that loss of fat mass with preservation of lean body mass decrease mortality [6].

Individuals also differ in their regional distribution of body fat, which have implications for their morbidity and mortality. Anthropometric measures of abdominal fatness (e.g. waist circumference (WC)) appears to be more strongly associated with the risk of type 2 diabetes, cardiovascular disease and mortality than anthropometric measures of general fatness (e.g. body mass index (BMI)) [7]. In particular, waist circumference (WC) adjusted for body mass index (BMI) is strongly and positively associated with mortality [8]–[12]. This has predominantly been attributed to accumulation of intra-abdominal fat [13]–[15]. In contrast, anthropometric measures of peripheral fatness (e.g. hip and thigh circumference) are associated with lower mortality [16]–[19] possibly due to favorable health effects of both the lean body mass [6]; [20]–[23] and the lower-body fat [24].

The association between mortality and changes in the localization of body fat is, however, not clear [25]. We therefore investigated the association between changes in WC and mortality in large cohort of healthy middle-aged men and women, and evaluated the influence from concurrent changes in BMI.

## Methods

In 1993–97, a random sample of 160,725 individuals aged 50–64 years were invited to the Danish prospective study ‘Diet, Cancer and Health’. A total of 57,053 accepted the invitation (569 were later excluded due to a cancer diagnosis, which was not, due to processing delays, registered in the Danish Cancer Registry at the time of the invitation). Participants filled in questionnaires and were clinically examined. In 1999–2002, repeated information was collected with questionnaires. The Danish Data protection Agency and the regional Ethical Committee approved the study, which was in accordance with the Helsinki Declaration II. Participants signed a written consent before participating. Details of the study are described elsewhere [26].

### Exposure measures

In 1993–97, technicians measured the individuals' height (nearest 0.5 cm without shoes) and weight (nearest 0.1 kg using a digital scale, with light clothes/underwear). The WC was measured (nearest 0.5 cm) with a measuring tape at the smallest horizontal circumference between the ribs and iliac crest (natural waist), or, in case of an indeterminable WC narrowing, halfway between the lower rib and the iliac crest. In 1999–02, individuals received a self-administrated questionnaire and reported their weight (kg) and WC (cm) measured at the level of the umbilicus using an enclosed paper measuring tape. BMI (kg/m2) was calculated as weight per height squared. Change in WC (DWC) (cm) and change in BMI (DBMI) (kg/m2) was calculated as the value in 1993–97 subtracted from the value in 1999–02.

The validity of the self-reported WC was assessed in study carried out in 408 men and women from the cohort [27]. A high correlation between the self-reported and technician measured WC was found, but there was some underreporting and rather wide limits of agreement in the comparison, and the circumferences were larger at the umbilicus than at the natural waist. The DWC was somewhat overestimated in women and slightly underestimated in men, and the difference was associated with baseline BMI (men) and WC (women). It was, however, concluded that the self-reported WC could be used as a proxy for the technician-measured WC in regression analyses of DWC if these were adjusted for baseline BMI and WC [27].

Covariates, assessed with the 1999–02 questionnaire, were used: *smoking habits* (never, ex, current smoker of <15 g/day, 15–25 g/day, >25 g/day), *sports activity* (0 versus >0 hours/wk) [28]; [29], *total energy intake (including alcohol)* (KJ/day) [28]; [30], diet quality assessed as a modified *Mediterranean diet score* [*31*], *drinking pattern* (abstainer, 0–3 times/month, 1–4 times/wk, 5–6 times/wk, daily), *educational level* (length of education: <8 years (basic school), 8–10 years (vocational education, higher education of 1–2 years), >10 years (vocational education, higher education of more than 2 years)) [26], and in women *menopausal status* (pre, post, unknown).

### Mortality

Information on all-cause mortality was obtained by linkage to the Danish Central Person Register using information about emigration, date of disappearance and vital status. The validity of all-cause mortality in the Danish Central Person Register is generally considered high [32].

### Exclusion criteria

Chronic disease may induce changes in anthropometry and increase the risk of early mortality [2]–[5]. We defined chronic disease according to a previously developed classification [33], and excluded individuals with diagnosed diseases occurring before examination in 1999–02 registered in the National Hospital Discharge Register that includes all hospitalisations since 1970 [34], and the The National Diabetes Register that includes individuals with diabetes treated at hospitals and in general practice since 1990 [35]; [36].

Men and women with extreme values on the anthropometric variables (values below the 0.5 and above the 99.5 sex-specific percentiles of WC and BMI, and below the 2.5 and above the 97.5 sex-specific percentiles DWC and DBMI) were also excluded due to potential measurement errors.

### Statistical Analyses

Analyses were conducted for each sex separately and sexes were combined when appropriate Hazard ratios (HR) of mortality were calculated from Cox proportional hazard models with years since the examination in 1999–02 as time axis, so that individuals were considered at risk from 1999–02 until time at death, emigration/disappearance or April 27 2008, whichever came first.

Analyzing continuous exposures in epidemiology has been widely debated [37], and we chose a strategy based on restricted cubic splines as these provide smooth curves that could be a plausible biological appearance for the investigated associations [37]. WC in 1993–97 was included as restricted cubic splines (3 knots) [38] in models with age in 1999–02 and years between examinations. Covariates were added in a second step, and BMI in 1993–97 was added in a third step. Similar analyses were conducted for BMI in 1993–97 with WC in 1993–97 added in the third step, and for WC and BMI measured in 1999–02. The DWC was included as restricted cubic splines (3 knots) [38] in models with age in 1999–02, years between examinations and WC in 1993–97. Covariates were added in a second step, and DBMI and BMI in 1993–97 were added in a third step. Similar analyses were conducted for DBMI with WC in 1993–97 and DWC added in the third step. Age in 1999–02, DBMI, WC and BMI in 1993–97 were included as restricted cubic splines (3 knots) [38]. Linearity of the remaining covariates was tested against a cubic spline, and was included as such, if non-linear associations were detected. A spline function was assumed to be significant if at least one of the splines differed significantly from zero assessed by an overall Wald test, and an association was assumed to be non-linear if the last splines differed significantly from zero assessed by Wald test. The proportional hazard assumption was assessed with log-rank test based on Schoenfeld residuals. No violations were detected.

### Subgroups Analyses

To explore if the association between DWC and mortality was equal throughout the range of the DBMI, the association between DWC and mortality were investigated in groups with loss (DBMI< = 0) and gain in BMI (DBMI>0). The association between DWC and mortality may also depend on the initial fatness level [39]. The association between DWC and mortality was therefore also investigated in groups with a high and low baseline WC (cut-off at the sex-specific median of WC (94 cm in men and 79 cm in women)). These differences were also formally tested on the multiplicative scale by cross-product terms using a Wald test.

Smoking and undiagnosed diseases may induce changes in anthropometry and increase the risk of mortality [2]–[5]. We explored this influence on the associations by exclusion of smokers and ex-smokers, and deaths occurring in the first one to seven years of follow-up.

Analyses were conducted in STATA version 9.2 (Stata Corporation, College Station, Texas; www.stata.com). Statistically significant differences were defined as differences with p<0.05.

## Results

Between the examinations in 1993–97 and 1999–02, 1778 individuals died and 460 emigrated/disappeared leaving 54,246 eligible for re-invitation. Among these, 5,865 did not respond, 2,858 did not want to participate, 649 had questionnaires with errors, and for 1,050 information on follow-up time, anthropometrics or covariates was missing leaving 20,667 men and 23,157 women. Among these, 6,759 men and 7,515 women were excluded due to a diagnose of chronic disease occurring before follow-up in 1999–02. Finally 1,324 men and 1,601 women were excluded due to extreme values on the anthropometric variables. Thus, 12,584 men and 14,041 women, who were presumed to be healthy, were eligible for the current study.

Table 1 provides the basic description of the cohort. The median observation time from the examination in 1999–02 to mortality or censoring was 6.7 years in men and 6.8 years in women. In this period, 568 and 361 deaths occurred among men and women, respectively. The median WC was 94 cm in men and 79 cm in women at baseline. During the 5.3 years between the two examinations, the median change in WC was 3 cm in men and 7 cm in women. In men, 3,833 (30%) had a loss in WC and 8,751 (70%) had a gain in WC. In women, 2,106 (15%) had a loss in WC and 11,935 (85%) had a gain in WC. The Pearson correlation between WC and BMI at baseline was high (0.83) in both sexes, but modest between DWC and DBMI in men (0.41) and women (0.36).

**Table 1:** Distribution of the study population by age and anthropometrics.

	Median (5–95%-tile)	
	Men (n = 12,584)	Women (n = 14,041)
Age (year) in 1993–97	55.4 (50.7∶64.0)	55.8 (50.7∶64.0)
Age (year) in 1999–02	60.8 (56.0∶69.3)	61.1 (56.0∶69.4
Time (year) between examinations in 1993–97 and in 1999–02	5.3 (5.0∶5.8)	5.3 (5.0∶5.9)
Time (year) between examination in 1999–02 and mortality/censoring	6.7 (5.8∶7.8)	6.8 (5.9∶7.8)
Body mass index (kg/m2) in 1993–97	25.7 (21.6∶31.2)	24.3 (20.0∶32.7)
Body mass index (kg/m2) in 1999–02	25.7 (21.6∶31.3)	24.2 (19.8∶31.6)
Changes in body mass index (kg/m2) between 1993–97 and 1999–02	0.0 (−1.8∶1.8)	−0.1 (−2.2∶2.0)
Waist circumference (cm) in 1993–97	94 (82∶109)	79 (67∶98)
Waist circumference (cm) in 1999–02	96 (85∶112)	86 (72∶107)
Changes in waist circumference (cm) between 1993–97 and 1999–02	3 (−5∶11)	7 (−3∶19)

### Baseline WC and BMI

**Figure 1:**
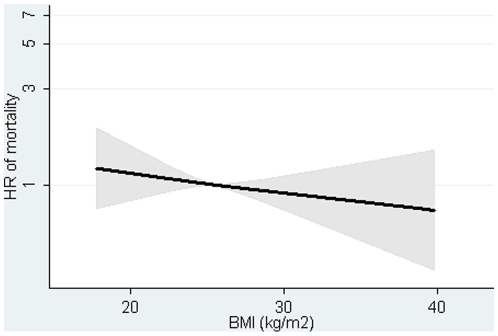
Hazard ratios and 95% confidence intervals of mortality according to body mass index (BMI) in 1993–97 with adjustment for waist circumference (WC). Lines are the hazard ratios (areas the 95%-confidence intervals) derived from Cox's proportional-hazard models where BMI was included as restricted cubic splines (3 knots). Reference point is the mean BMI. Years since the examination in 1999–02 was used as underlying time axis. Adjusted for: sex, years between examinations, age in 1999–02, BMI in 1993–97, smoking habits, Mediterranean diet score, energy intake, education, drinking pattern, sports activity and menopausal status (women only). Test of linearity p = 0.9147 (linear association). Test of effect p = 0.4956.

**Figure 2:**
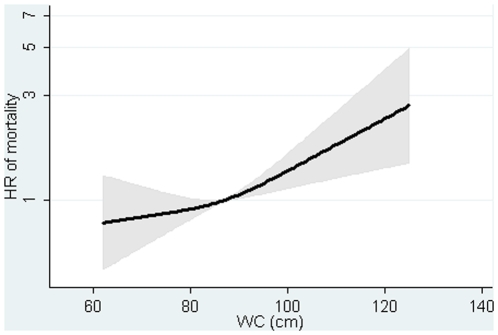
Hazard ratios and 95% confidence intervals of mortality according to waist circumference (WC) in 1993–97 with adjustment for body mass index (BMI). Lines are the hazard ratios (areas the 95%-confidence intervals) derived from Cox's proportional-hazard models where WC was included as restricted cubic splines (3 knots). Reference point is the mean WC. Years since the examination in 1999–02 was used as underlying time axis. Lines are adjusted for: sex, years between examinations, age in 1999–02, BMI in 1993–97, smoking habits, Mediterranean diet score, energy intake, education, drinking pattern, sports activity and menopausal status (women only). Test of linearity p = 0.1704 (linear association). Test of effect p = 0.0046.

The association between BMI at baseline and mortality was positive in men and women with adjustment for covariates, but inverse and weak after additional adjustment for WC (Table 2, Figures S1–S2). The HR was 0.98 (0.94, 1.01) per one kg/m2 for the sexes combined after adjustment for covariates and WC (Table 2, Figure 1). The association between baseline WC and mortality was positive in men and women (Table 2, Figures S3–S4). The HR was 1.11 (1.04, 1.18) per 5 cm in the sexes combined after adjusting for covariates and BMI. Similar results were found for BMI and WC measured at follow-up (Table 2, Figure 2).

**Table 2:** Hazard ratios and 95% confidence intervals of mortality according to body mass index (BMI) and waist circumference (WC) and in 1993–97 and 1999–02.

	Crude	Adjusted	Adjusted + WC	Adjusted + BMI
**1993–97**	HR (95% CI)*	**HR (95% CI)** * †	HR (95% CI)* † ‡	HR (95% CI) * † ‡
BMI (kg/m2)	1.02 (1.01, 1.05)	1.03 (1.00, 1.07)	0.98 (0.94, 1.01)	–
BMI in men (kg/m2)	1.02 (1.00, 1.05)	1.03 (1.00, 1.06)	0.97 (0.92, 1.02)	–
BMI in women (kg/m2)	1.01 (0.98, 1.05)	1.01 (0.98, 1.04)	0.98 (0.93, 1.04)	–
WC (5 cm)	1.13 (1.10, 1.17)	1.07 (1.03, 1.11)	–	1.11 (1.04, 1.18)
WC in men (5 cm)	1.13 (1.03, 1.24)	1.09 (1.03, 1.14)	–	1.10 (0.98, 1.23)
WC in women (5 cm)	1.06 (1.01, 1.12)	1.04 (0.94, 1.10)	–	1.06 (0.97, 1.18)
**1999–02**				
BMI (kg/m2)	1.07 (1.00, 1.14)	1.01 (0.99, 1.04)	0.97 (0.94, 1.04)	–
BMI in men (kg/m2)	1.01 (0.98, 1.04)	1.02 (0.99, 1.04)	0.97 (0.94, 1.00)	–
BMI in women (kg/m2)	1.01 (0.98, 1.04)	1.01 (0.98, 1.03)	0.97 (0.93, 1.01)	–
WC (5 cm)	1.12 (1.09, 1.16)	1.07 (1.03, 1.10)	–	1.11 (1.05, 1.17)
WC in men (5 cm)	1.08 (1.03, 1.13) §	1.07 (1.02, 1.13)	–	1.12 (1.04, 1.21)
WC in women (5 cm)	1.09 (1.02, 1.17)	1.05 (1.00, 1.10)	–	1.07 (1.02, 1.12)

*Adjusted for years between examinations, age in 1999–02 and sex in combined analyses.

† Adjusted for smoking habits, Mediterranean diet score, energy intake, education, drinking pattern, sports activity and menopausal status (women only).

‡ WC added to analyses of BMI, and BMI added to analyses of WC.

All associations were accepted to be linear, except §.

### Changes in WC and Changes in BMI

**Figure 3:**
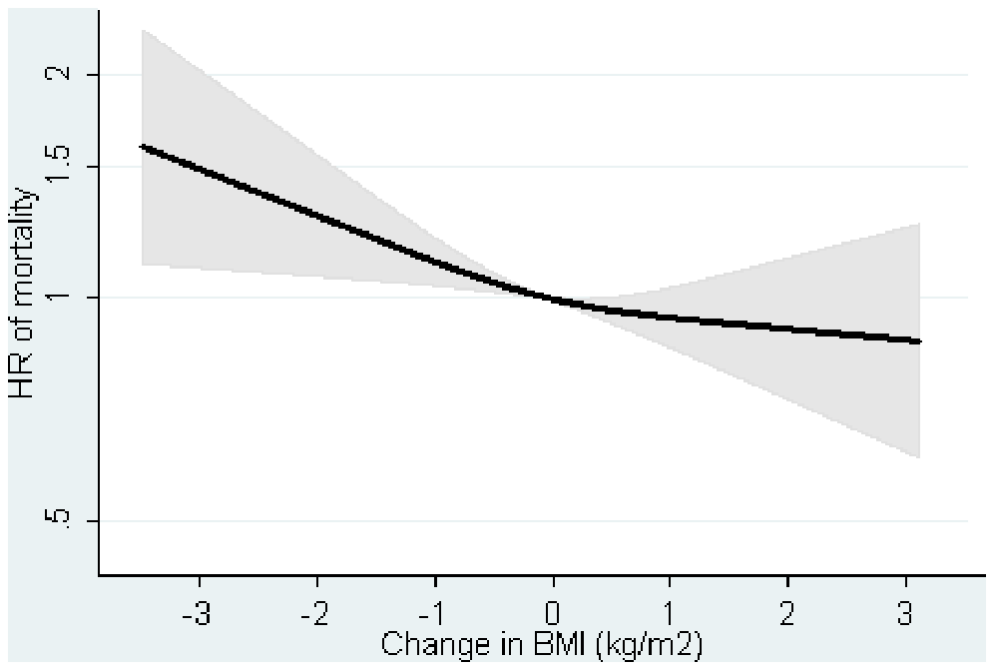
Hazard ratios and 95% confidence intervals of mortality according to changes in body mass index (DBMI) with adjustment for changes in waist circumference (DWC). Lines are the hazard ratio (areas the 95%-confidence intervals) derived from Cox's proportional-hazard models where DBMI was included as restricted cubic splines (3 knots). Reference point is the mean DBMI. Years since the examination in 1999–02 is the underlying time axis. Adjusted for: sex, years between examinations, age in 1999–02, baseline BMI, baseline WC, DWC, smoking habits, Mediterranean diet score, energy intake, education, drinking pattern, sports activity and menopausal status (women only). Test of linearity p = 0.3159 (linear association). Test of effect p = 0.0074.

**Figure 4:**
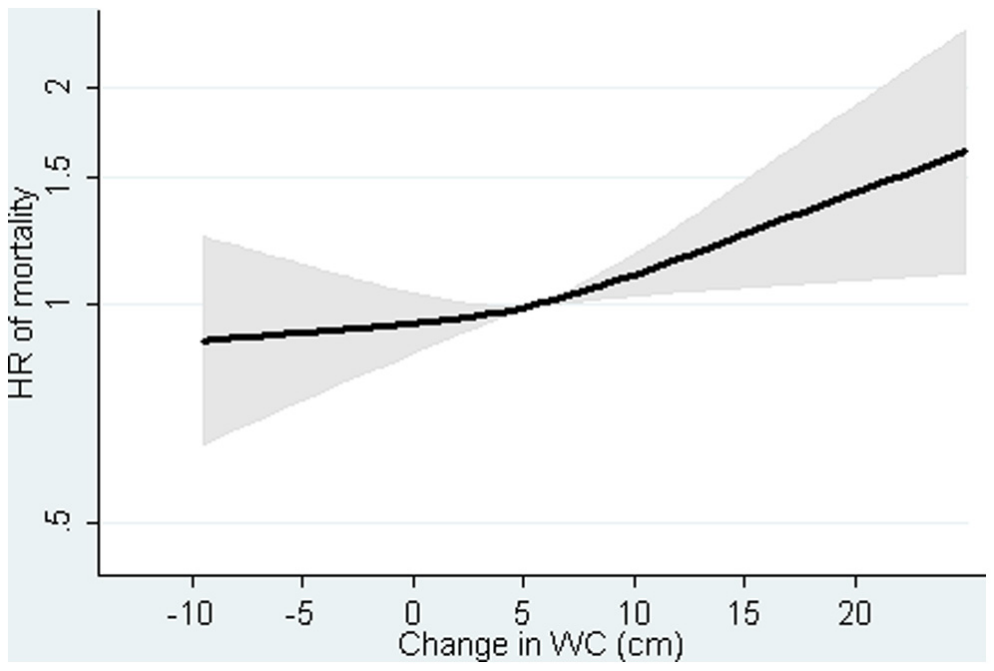
Hazard ratios and 95% confidence intervals of mortality according to changes in waist circumference (DWC) with adjustment for changes in body mass index (DBMI). Lines are the hazard ratio (areas the 95%-confidence intervals) derived from Cox's proportional-hazard models where DWC was included as restricted cubic splines (3 knots). Reference point is the mean DWC. Years since the examination in 1999–02 is the underlying time axis. Adjusted for: sex, years between examinations, age in 1999–02, baseline BMI, baseline WC, DBMI, smoking habits, Mediterranean diet score, energy intake, education, drinking pattern, sports activity and menopausal status (women only). Test of linearity p = 0.3079 (linear association). Test of effect p = 0.0153.

The DBMI was inversely associated with mortality in men and women (Table 3, and Figures S5–S6). The HR was 0.91 (0.86, 0.97) per kg/m2 for the sexes combined after adjusting for covariates baseline BMI, WC and DWC (Table 3, Figure 3). The DWC was positively associated with mortality in men and women (Table 3, and Figures S7–S8). The HR was 1.09 (1.02, 1,16) per 5 cm for the sexes combined after adjusting for covariates baseline BMI, WC and DBMI (Table 3, Figure 4).

**Table 3:** Hazard ratios and 95% confidence intervals of mortality according to changes in body mass index (DBMI) and changes in waist circumference (DWC).

	Crude	Adjusted	Adjusted + DWC	Adjusted + DBMI
	HR (95% CI)*	HR (95% CI)* †	HR (95% CI)* † ‡	HR (95% CI) * † ‡
DBMI (kg/m2)	0.95 (0.90, 1.01)	0.94 (0.90, 1.00)	0.91 (0.86, 0.97)	–
DBMI in men (kg/m2)	0.89 (0.82, 0.97) §	0.90 (0.84, 0.98)	0.88 (0.80, 0.95)	–
DBMI in women (kg/m2)	0.98 (0.90, 1.07)	0.98 (0.90, 1.06)	0.95 (0.87, 1.04)	–
DWC (5 cm)	1.02 (0.96, 1.10)	1.04 (0.98, 1.10)	–	1.09 (1.02, 1.16)
DWC in men (5 cm)	1.00 (0.92, 1.09)	1.01 (0.92, 1.09)	–	1.08 (0.98, 1.18)
DWC in women (5 cm)	1.10 (1.00, 1.17)	1.06 (0.98, 1.16)	–	1.09 (1.00, 1.18)

*Adjusted for years between examinations, age in 1999–02, baseline BMI (analyses of DBMI), baseline WC (analyses of DWC) and sex in the combined analyses.

† Adjusted for smoking habits, Mediterranean diet score, energy intake, education, drinking pattern, sports activity, menopausal status (women only).

‡ DWC and baseline WC added to analyses of DBMI and DBMI and baseline BMI added to analyses of DWC.

All associations were accepted to be linear, except §.

### Subgroup Analyses

The association between DWC and mortality was positive in both strata of DBMI. The HR was 1.15 (1.06, 1.26) per 5 cm for in participants with concurrent loss of BMI and 1.02 (0.92, 1.12) per 5 cm in participants with concurrent gain in BMI after adjusting for covariates baseline BMI, WC and DBMI (p for interaction  = 0.06).

The DWC was also positively associated with mortality in the two strata of baseline WC. The HR was 1.07 (0.97, 1.18) per 5 cm for in participants with low baseline WC and 1.11 (1.02, 1.20) per 5 cm in participants with high baseline WC after adjusting for covariates baseline BMI, WC and DBMI (p for interaction  = 0.31).

Exclusion of current smokers and ex-smokers or deaths occurring within the first years of follow-up had no major influence on the associations between DWC, DBMI and mortality (Table 4).

**Table 4:** Hazard ratios and 95% confidence intervals of mortality according to changes in waist circumference (DWC) and changes in body mass index (DBMI) when smokers or deaths occurring in the first years of follow-up are excluded.

	HR (95% CI)*	HR (95% CI)*	HR (95% CI)*	HR (95% CI)*	HR (95% CI)*
	Non-smokers	1 y	3 y	5 y	7 y
Cases	n = 199	n = 884	n = 655	n = 361	n = 49
DBMI (kg/m2)	0.90 (0.78, 1.03)	0.91 (0.85, 0.97)	0.92 (0.86, 0.99)	0.93 (0.84, 1.03)	0.73 (0.57, 0.95)
DWC (kg/m2)	1.12 (0.99, 1.27)	1.09 (1.03, 1.17)	1.11 (1.03, 1.19)	1.05 (0.95, 1.17)	1.04 (0.80, 1.68)

*Adjusted for, sex, years between examinations, age in 1999–02, baseline BMI, baseline WC, DWC (analyses of DBMI), DBMI (analyses of DWC), smoking habits, Mediterranean diet score, energy intake, education, drinking pattern, sports activity and menopausal status (women only).

All associations were accepted to be linear.

## Discussion

This prospective study of healthy middle-aged men and women showed that changes in WC were positively associated with mortality, whereas changes in BMI were inversely associated with mortality. The positive association with changes in WC was stronger after adjustment for concurrent changes in BMI, and the inverse association with changes in BMI was stronger after adjustment for concurrent changes in WC.

The strengths of the study are the large-scale, well-characterized study population with anthropometry and covariates assessed at two subsequent time points and the complete follow-up (99.5%). Despite this large study population, there were relatively few deaths, especially among those with loss of WC.

In total, 57,053 (36%) of the invited individuals participated at baseline [26], and we excluded many of these due to missing data or predefined exclusion criteria leaving 26,625 individuals in the current study. Our participants were younger, better educated, had higher WC and BMI, a healthier lifestyle and were less diseased than non-participants (Table S1). This selective study population, may have minimized the risk of confounding or modification from known or unknown risk factors, but also restricted the generalization of the results to populations of fairly healthy middle-aged individuals with a healthier than average lifestyle.

Chronic disease may induce changes in anthropometry [2]–[5], and we aimed to effectively eliminate this influence by excluding individuals with a wide range of chronic diseases [33] that were diagnosed before and during the waist change period. The registers used to identify these individuals are fairly complete and valid [34]; [36]. Individuals with undiagnosed/sub-clinical diseases or various psychiatric diseases (e.g. depression) are, however, not identified by these registers. We can therefore not definitely exclude influence from underlying diseases on the associations. We do, however, find it unlikely that several diseased individuals would participate in a long-term cohort study, which is supported by lower morbidity and mortality in the cohort compared with the general Danish population [26]. Furthermore, we did not find an increased mortality in those with loss of WC and exclusion of smokers and deaths occurring within the first years of follow-up had no notable influence on the associations. This suggests that the influence from diseases on this association was reduced, or even eliminated, by the exclusions. We may, however, speculate that individuals with clinically manifest or sub-clinical diseases are those who are most susceptible to changes in anthropometry [2]–[5], whereby our extensive exclusion have minimized the risk of bias, but also the ability to identify strong health effects.

Covariates that could have confounding or modifying effects (age, smoking, physical activity, diet, alcohol, education and menopausal status) were included in the study, but had no notable effects on the direction and strength of the associations. Some residual confounding from these or unmeasured covariates could, however, still be present. It has been argued that a distinction between intentional and unintentional weight loss is important to handle the influence from underlying diseases in population based studies of changes in anthropometry [2]–[5]. We had no such information, but the distinction may be artificial [40], and our extensive exclusion of diseased individuals by the use of the unique Danish registers may be more valid way to reduce the influence from diseases.

Different measurement methods of WC and BMI were employed in 1993–97 and 1999–02. A validation study within the cohort [27] found that the two measures could be used together in analyses of changes in WC if these were adjusted for baseline BMI and WC [27]. We adjusted for baseline BMI and WC to assure that potential factors associated with selective misreporting and mortality was captured by this adjustment. We also excluded individuals with extreme anthropometric measurements as selective misreporting may be most pronounced in these individuals. The exclusions provided more consistent and precise results, but we may have restricted the generalization of the results, and still we cannot exclude that some degree of measurement error persist.

Long-term population-based studies have shown increased mortality in healthy individuals who loose weight [2]–[5]. This was also observed in the current study, as changes in BMI was inversely associated with mortality, in particular after adjustment for concurrent changes in WC. Weight loss is, however, composed of losses of fat and lean body mass, and the various fat compartments and the lean body mass have different impact on mortality [2]; [6]–[13]; [16]–[23]. The health effects of weight loss may thus reflect a balance between losses of harmful abdominal fat versus losses of beneficial peripheral subcutaneous fat and lean body mass [2]. Individuals may thus benefit from a weight loss that selectively reduces the abdominal fat, but if such weight loss also reduces the lean body mass and the peripheral subcutaneous fat, then the beneficial health effects may become outweighed. To investigate if beneficial health effects of weight loss depended on loss of abdominal fat, we examined the association between changes in WC and mortality in healthy middle-aged men and women. The direction of the association was compatible with this hypothesis as a 5 cm loss of WC was associated with a 9% lower risk of mortality, whereas one kg/m2 loss of BMI was associated with a 9% higher risk of mortality. Thus, by focusing on changes in WC, the expected effects of loss and gain were found. Changes in WC were also positively associated with the risk of mortality from coronary heart disease in postmenopausal women with established heart disease in the Heart and Estrogen/Progestin Replacement Study [10], but only among women assigned to hormone therapy who were in the extreme five percent of the waist change distribution. Estimates for overall weight change were, however, not shown, although changes in weight were inversely associated with the risk of mortality from coronary heart disease [10].

Recent studies, including studies within the current population [8]; [12], have shown that WC adjusted for BMI is strongly and positively associated with mortality [8]–[12]. The mechanisms that explain this association are not firmly established, but it has been suggested that WC adjusted for BMI acts as a surrogate measure for intra-abdominal fatness [8]; [9]; [41]. The positive association between changes in WC and mortality was slightly stronger after adjustment for concurrent changes in BMI. The adjustment may reduce confounding, but does also introduce a substitution aspect in the interpretation of the results. The higher risk of mortality associated with gain in WC may e.g. be explained by gain in harmful abdominal fat or by loss of beneficial peripheral fat or lean body mass that may accompany the WC gain as changes in BMI are fixed. These effects cannot be directly separated from the results, but underscore that redistribution of fat mass towards the abdominal region is a risk factor for mortality.

In conclusion, changes in WC are positively associated with all-cause mortality in healthy middle-aged men and women throughout the range of concurrent changes in BMI. These findings suggest a need for development of prevention and treatment strategies targeted against redistribution of fat mass towards the abdominal region.

## Supporting Information

Figure S1 Hazard ratios and 95% confidence intervals of mortality according to body mass index (BMI) in 1993–97 with adjustment for waist circumference (WC) in men. Lines are the hazard ratios (areas the 95%-confidence intervals) derived from Cox's proportional hazard models where BMI was included as restricted cubic splines (3 knots). Reference points are the respective means of BMI. Years since the examination in 1999–02 was used as underlying time axis. Adjusted for: years between examinations, age in 1999–02, BMI in 1993–97, smoking habits, Mediterranean diet score, energy intake, education, drinking pattern and sports activity. Test of linearity p = 0.3059 (linear association). Test of effect p = 0.3048.
(0.01 MB TIF)

Figure S2 Hazard ratios and 95% confidence intervals of mortality according to body mass index (BMI) in 1993–97 with adjustment for waist circumference (WC) in women. Lines are the hazard ratios (areas the 95%-confidence intervals) derived from Cox's proportional hazard models where BMI was included as restricted cubic splines (3 knots). Reference points are the respective means of BMI. Years since the examination in 1999–02 was used as underlying time axis. Adjusted for: years between examinations, age in 1999–02, BMI in 1993–97, smoking habits, Mediterranean diet score, energy intake, education, drinking pattern, sports activity and menopausal status. Test of linearity p = 0.4510 (linear association). Test of effect p = 0.6572.
(0.01 MB TIF)

Figure S3 Hazard ratios and 95% confidence intervals of mortality according to waist circumference (WC) in 1993–97 with adjustment for body mass index (BMI) in men. Lines are the hazard ratios (areas the 95%-confidence intervals) derived from Cox's proportional hazard models where WC was included as restricted cubic splines (3 knots). Reference points are the respective means of WC. Years since the examination in 1999–02 was used as underlying time axis. Lines are adjusted for: years between examinations, age in 1999–02, BMI in 1993–97, smoking habits, Mediterranean diet score, energy intake, education, drinking pattern and sports activity. Test of linearity p = 0.0603 (linear association). Test of effect p = 0.0041.
(0.01 MB TIF)

Figure S4 Hazard ratios and 95% confidence intervals of mortality according to waist circumference (WC) in 1993–97 with adjustment for body mass index (BMI) in women. Lines are the hazard ratios (areas the 95%-confidence intervals) derived from Cox's proportional hazard models where WC was included as restricted cubic splines (3 knots). Reference points are the respective means of WC. Years since the examination in 1999–02 was used as underlying time axis. Lines are adjusted for: years between examinations, age in 1999–02, BMI in 1993–97, smoking habits, Mediterranean diet score, energy intake, education, drinking pattern, sports activity and menopausal status. Test of linearity p = 0.8246 (linear association). Test of effect p = 0.4041.
(0.01 MB TIF)

Figure S5 Hazard ratios and 95% confidence intervals of mortality according to changes in body mass index (DBMI) with adjustment for changes in waist circumference (DWC) in men. Lines are the hazard ratio (areas the 95%-confidence intervals) derived from Cox's proportional hazard models where DBMI was included as restricted cubic splines (3 knots). Reference points are the respective means of DBMI. Years since the examination in 1999–02 is the underlying time axis. Adjusted for: years between examinations, age in 1999–02, baseline BMI, baseline WC, DWC, smoking habits, Mediterranean diet score, energy intake, education, drinking pattern and sports activity. Test of linearity p = 0.2261 (linear association). Test of effect p = 0.0041.
(0.01 MB TIF)

Figure S6 Hazard ratios and 95% confidence intervals of mortality according to changes in body mass index (DBMI) with adjustment for changes in waist circumference (DWC) in women. Lines are the hazard ratio (areas the 95%-confidence intervals) derived from Cox's proportional hazard models where DBMI was included as restricted cubic splines (3 knots). Reference points are the respective means of DBMI. Years since the examination in 1999–02 is the underlying time axis. Adjusted for: years between examinations, age in 1999–02, baseline BMI, baseline WC, DWC, smoking habits, Mediterranean diet score, energy intake, education, drinking pattern, sports activity and menopausal status. Test of linearity p = 0.7999 (linear association). Test of effect p = 0.5332.
(0.01 MB TIF)

Figure S7 Hazard ratios and 95% confidence intervals of mortality according to changes in waist circumference (DWC) with adjustment for changes in body mass index (DBMI) in men. Lines are the hazard ratio (area the 95%-confidence intervals) derived from Cox's proportional hazard models where DWC was included as restricted cubic splines (3 knots). Reference points are the respective means of DWC. Years since the examination in 1999–02 is the underlying time axis. Adjusted for: years between examinations, age in 1999–02, baseline BMI, baseline WC, DBMI, smoking habits, Mediterranean diet score, energy intake, education, drinking pattern and sports activity. Test of linearity p = 0.5820 (linear association). Test of effect p = 0.2808.
(0.01 MB TIF)

Figure S8 Hazard ratios and 95% confidence intervals of mortality according to changes in waist circumference (DWC) with adjustment for changes in body mass index (DBMI) in women. Lines are the hazard ratio (areas the 95%-confidence intervals) derived from Cox's proportional-hazard models where DWC was included as restricted cubic splines (3 knots). Reference points are the respective means of DWC. Years since the examination in 1999–02 is the underlying time axis. Adjusted for: years between examinations, age in 1999–02, baseline BMI, baseline WC, DBMI, smoking habits, Mediterranean diet score, energy intake, education, drinking pattern, sports activity and menopausal status. Test of linearity p = 0.5374 (linear association). Test of effect p = 0.1131.
(0.01 MB TIF)

Table S1 Distribution of participants (n = 26,625) and excluded (n = 30,428) according to baseline characteristics.
(0.03 MB DOC)
