# Supplementary material for: Changes in Waist Circumference and Mortality in Middle-Aged Men and Women
Source: PLoS One. 2010 Sep 30;5(9):e13097. doi: 10.1371/journal.pone.0013097 (PMC2948031; doi:10.1371/journal.pone.0013097)
Supplement: Table S1 — Distribution of participants (n = 26,625) and excluded (n = 30,428) according to baseline characteristics. (0.03 MB DOC) [file pone.0013097.s009.doc]

#### **Table S1. Distribution of participants (n=26,625) and excluded (n=30,428)** according to baseline characteristics

|  | **Men** | | **Women** | |
| --- | --- | --- | --- | --- |
|  | **Participants** | **Excluded** | **Participants** | **Excluded** |
|  | **Median (5-95%-tile)** | **Median (5-95%-tile)** | **Median (5-95%-tile)** | **Median (5-95%-tile)** |
| Age (y) in 1993-97 | 55.4 (50.7, 64.0) | 56.6 (50.8, 64.3) | 55.8(50.7, 64.0) | 56.8 (50.8, 64.4) |
| WC (cm) in 1993-97 | 94 (82, 109) | 97 (81, 117) | 79 (67, 98) | 82 (67, 107) |
| BMI (kg/m^2^) in 1993-97 | 25.7 (21.6, 31.2) | 26.7 (21.3, 34.3) | 24.3 (20.0, 32.7) | 25.4 (19.7, 35.5) |
| Mediterranean Diet | 5 (2, 7) | 4 (2, 7) | 5 (2, 7) | 4 (2, 7) |
| Energy Intake (Mj./d) | 10.8 (7.2, 15.7) | 10.7 (7.0, 16.1) | 8.6 (5.6, 12.6) | 8.5 (5.3, 12.9) |
| Chronic diseased | 0% | 58% | 0 % | 59% |
| Current smokers | 34% | 44% | 27% | 38% |
| Physical inactive | 45% | 57% | 36% | 47% |
| Alcohol abstainers | 1% | 3% | 2% | 4% |
| Less than 8 y of school | 30% | 39% | 27% | 36% |
| Postmenopausal | - | - | 57% | 60% |

Covariates assessed in 1993-97 are used. # Diagnosed chronic disease (defined in reference 28), and occurring before follow-up in 1999-02.
